# Test–Retest Reliability and Sensitivity of Common Strength and Power Tests over a Period of 9 Weeks

**DOI:** 10.3390/sports10110171

**Published:** 2022-11-02

**Authors:** Maria Venegas-Carro, Andreas Kramer, Maria Moreno-Villanueva, Markus Gruber

**Affiliations:** Human Performance Research Centre, Department of Sport Science, University of Konstanz, 78464 Konstanz, Germany

**Keywords:** countermovement-jump, CMJ, handgrip strength, knee extension, MVC, reactive hop, reliability, sensitivity

## Abstract

This study evaluated the reliability and sensitivity of a set of different common strength and power tests in a healthy adult population in a span of 9 weeks. Seventeen subjects (24.2 ± 2.2 years, 1.75 ± 0.10 m, 68.6 ± 14.2 kg, seven women) participated in the study. We tested countermovement jumps, reactive hops, and the maximal voluntary contraction (MVC) of handgrip and isometric knee extension. The tests were conducted in three separate sessions across a nine-week period, with one week between the first two sessions and eight weeks between the second and the third. Reliability and sensitivity statistics for each test were calculated for both the average of three trials and the best result during each session. The MVC of isometric knee extension and handgrip, as well as the countermovement jump test, demonstrated very high reliability and sensitivity over the nine-week period. The peak force of the reactive hops demonstrated high reliability but high sensitivity only for the average but not for the best result. The average contact time of reactive hops was neither a sensitive nor reliable measurement. In conclusion, isometric maximal knee extension and handgrip tests, as well as countermovement jumps and peak force of reactive hops, can be used as reliable and sensitive measurements of isometric and reactive strength and power over time periods of up to eight weeks. We recommend the use of the average results of three trials instead of the best performance value for longitudinal studies, as this procedure produces more consistent results and a lower measurement error.

## 1. Introduction

A major goal in the field of exercise science is to evaluate the degree to which training affects performance. Whether being an intervention study (e.g., measuring changes between repeated measurements), a training protocol with an athlete or a team (e.g., tracking changes in response to training or monitoring the training load), or measuring functional ability in older adults, to properly measure and evaluate this impact a variety of performance tests are commonly used with the test–retest method. Therefore, as sports scientists, coaches, physicians, or trainers, we rely on empirical data for comparisons and conclusions. To ensure that the interpretation of the results and inferences drawn from these data are correct, it is important to perform accurate measurements that are highly reproducible, that the tests are able to detect small changes in performance, and that the changes found in performance are real and not due to error or measurement noise [1].

The reliability of a test tells us the level of reproducibility and consistency between two or more measurements [2]. Any observed score of a measurement is composed of the sum of the true score plus an error component (i.e., measurement error). The estimate between how much of the result belongs to the error and how much to the accurate reading represents the measure of reliability [3,4]. In other words, the smaller the error, the better or more reliable the measurement [2]. Depending on the source of the measurement error, there are different types of reliability. In the context of this paper, reliability refers to stability or test–retest reliability, which is the variability of a test over time [5]. Furthermore, determining the sensitivity of a test tells us if it is able to detect meaningful and small changes between measurements [6].

Physical performance tests are commonly used in the medical and exercise science environment. They allow different aspects of the physical and sporting state of a person to be measured in a controlled and scientific manner [6]. For this, depending on the purpose and intended use, a wide range of tests are available. Specifically, neuromuscular performance is usually measured by strength and power tests. For example, the countermovement jump test (CMJ) has been thoroughly used in exercise testing in different settings to measure the explosive power of the lower limb [7,8,9], with which one can additionally monitor the neuromuscular fatigue [10], muscle strength [11], and training load [12] in athletes or subjects of different situations. Reactive hopping, a type of jumping that relies on the stretch-shortening cycle (SSC), is commonly used not only to measure the leg’s reactive force but also to calculate the leg or vertical stiffness [13,14] which are key factors for performance in sprinting [15,16,17], running [18], and other jumping types of sports [19]. Moreover, the maximal voluntary contraction (MVC) of the handgrip (HG) and knee extension (KE) is commonly used in different populations to measure and characterize the overall upper and lower extremity muscle strength [20,21]. HG strength is a particularly important reference measurement in older populations to identify a poor health status [22].

Test–retest reliability and sensitivity have been proven to be excellent for all these tests over a period of days or one week [7,23,24,25,26,27,28,29,30,31,32,33,34]. However, to the best of our knowledge, only a few studies have implemented intervals of more than one week between measurements to test reliability and sensitivity, although many longitudinal training or intervention studies run over a period of 6 to 12 weeks. To date, there are no studies for the MVC of the KE and reactive hops that have included a period longer than one week. For the CMJ, Moir et al. [32] included four sessions, each one week apart, and made comparisons between all sessions. Arteaga et al. [23] tested six sessions with an interval of 10 to 15 days between measurements but calculated reliability as the pooled value of all sessions together and did not compare them. For the MVC of the HG, Hogrel [30] tested two sessions with an average interval of 31 days but varied between participants from 1 to 90 days. Moreover, Bohannon and Schaubert [24] measured community-dwelling elders two times in a single-trial, 12 weeks apart. Therefore, in the present study, we applied a realistic and consistent interval between measurements that could be translated into the applied field. Moreover, in order to determine whether the use of the average or the best performance value of trials in a session produces more accurate and reproducible results, we made comparisons between these two values for all tests. In other words, the aim of this study was to evaluate the reliability and sensitivity of a set of different common strength and power tests in a healthy adult population over a span of nine weeks.

## 2. Materials and Methods

### 2.1. Study Design

**Figure 1:**
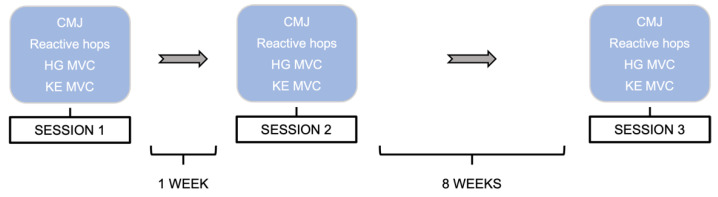
Schematic representation of the study sessions. CMJ: countermovement jumps; HG: handgrip; MVC: maximal voluntary contraction; KE: knee extension.

This study comprised a single-group, longitudinal design, whereby a series of performance measurements were tested in three separate sessions across a 9-week period (S1, S2, and S3). The first and second sessions were separated by 1 week, and the second and third by 8 weeks (see Figure 1 for the detailed study design). These two intervals were selected with the intention that (1) there would be enough time between the first and the second session (1 week) to avoid any training effect, and (2) the interval between the first and third sessions and the second and third sessions resembles a credible time normally found in training interventions (8–12 weeks). Additionally, the idea of having two initial sessions was intended to determine whether a familiarization session is required for any of the tests. The study was conducted at the Human Performance Research Centre, University of Konstanz, Germany. In each session, subjects performed 3 CMJ, 2 × 10 reactive hops, 3 HG MVC, and 3 isometric KE MVC with 1–2 min rest between repetitions depending on the test. The order in which the different tests were performed was randomly set for every participant and repeated in the same order for each of the three sessions. The time of the session at which each participant performed the tests during the first visit was kept for the following two sessions. Prior to the start of the tests, the participant’s height and weight were measured, and they performed a standardized warm-up consisting of 3 bodyweight squats, 10 heel raises, 3 submaximal CMJ, and 10 submaximal reactive hops. Participants were asked to maintain their usual level and kind of sports activities as constant as possible for the duration of the whole study and to avoid strenuous activities for the two days before every testing session.

### 2.2. Subjects

Seventeen healthy and recreationally active subjects (24.2 ± 2.2 years, 7 females and 10 males; see Table 1 for more details) participated in the study. An inclusion criterium was age ranging from 18–55 years. Exclusion criteria were (1) bone fracture(s) during the past twelve months, (2) injuries of the lower extremities during the six months before the start of the study, (3) heart problems, or (4) body mass index >30.

### 2.3. Isometric Leg Strength

**Figure 2:**
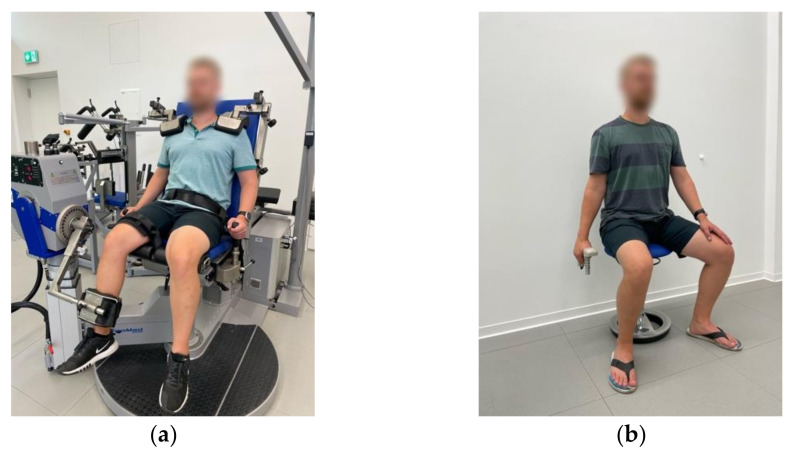
Correct positioning of participants during maximal voluntary contractions (MVC). (**a**) Knee extension MVC of the right leg with IsoMed-2000 dynamometer; (**b**) handgrip MVC of the right hand with Jamar^®^ dynamometer.

The KE isometric MVC was recorded unilaterally (right side) in the IsoMed-2000 dynamometer (D&R Ferstl GmbH, Hemau, Germany). The test was performed in a sitting position with the hip and knee joints at 90° and 60° of flexion, respectively, and the popliteal fossa of the tested leg touching the frontal edge of the seat. The dynamometer’s lever arm’s shin pad was secured to the participant’s right leg, 3 cm above the lateral malleolus. Adjustable straps and pads on the shoulders, hip, and right femur were used to minimize extraneous body movements. During contraction, participants were allowed to grip the side handles of the equipment situated at both sides of the hip. The knee’s anatomical axis of rotation was matched with the dynamometer’s mechanical axis, with the right lateral femoral epicondyle as a reference point (for a detailed image of the position, see Figure 2a). In all sessions as well as for all data acquisition and resulting peak torques, the manufacturer’s integrated computer software IsoMed Analyze V.1.0.5 (D&R Ferstl GmbH, Hemau, Germany) was used. Each participant’s position and settings on the dynamometer were recorded with the same software during the first session and were replicated throughout the rest of the sessions to ensure similar conditions across the whole test period. After the subject was completely fixed on the dynamometer, gravity adjustment of the IsoMed Analyzesoftware was applied by taking into consideration the weight of the tested leg in a resting position. Prior to testing, subjects performed a warm-up consisting of six submaximal ramp-and-hold contractions, with increasing intensity, separated by 30 s each. A 1 min break separated the warm-up and the testing. MVC tests consisted of three repetitions of about 3 s, during which the subjects were instructed to contract their muscles “as hard as possible”. During each trial, strong verbal encouragement and visual online feedback were provided to ensure maximal effort. A 2 min rest period was interspersed between repetitions. Torque data (Nm) were sampled at 2 kHz, and the peak torque of every trial was extracted and saved for further analysis as the mean of all trials (Avg) and the highest value of all three (Hv).

### 2.4. Handgrip Strength

Handgrip MVC was measured three times during each session with the Jamar^®^ hand-grip dynamometer (Jamar Plus+, Performance Health UK Ltd., Sutton-in-Ashfield, UK). Measurements were performed in the preferred hand of each participant. The preferred hand was selected by participants during the first session and did not necessarily match the dominant hand. The position for this measurement involved sitting down with the non-preferred hand on the thigh and the preferred hand holding the device and hanging down at the side of the body (elbow extended 180°). For a detailed image of the position, see Figure 2b. Instruction was to “squeeze the device as strong as possible for 2–3 s”. Before the first trial on the first session, each participant was allowed to choose the preferred or more comfortable handle position in the instrument (4 different positions available), and the chosen one was kept constant for all tests during all sessions. Verbal encouragement was given in every trial to produce the maximal possible effort. The maximal value (kg) for every trial was recorded and saved for statistical analysis as the mean of the three trials per session (Avg) and the highest value of each session (Hv).

### 2.5. Countermovement Jumps

This test consisted of three maximal CMJ on a force plate (Leonardo GRFP, Novotec medical GmbH, Pforzheim, Germany). Before the first test, all participants were shown and practiced the correct execution of the jump. They were asked to “quickly drop to a half-squat position and then immediately jump as high as possible” (with hands akimbo). A 1-min of rest was given between each jump. Data acquisition and analysis for all tests on the force plate (i.e., reactive hops, CMJ) were performed with Leonardo Mechanography software (version 4.3b01.93, Novotec Medical GmbH, Pforzheim, Germany) and barefoot on the same force plate. Ground reaction forces were sampled and recorded at 800 Hz. The maximal jump height (cm) and jump peak power (W/kg) were extracted and used for further analyses as the mean of all three jumps (Avg) and the highest height or the highest peak power value of the session (Hv).

### 2.6. Reactive Hops

Two sets of 10 bilateral reactive hops were performed on a force plate. Before the first test, all subjects were shown and practiced the correct execution of the hops. The instructions were “jump as stiff as possible, while still jumping as high as the stiffness allows; do not let the heels touch the plate during landing, keep the contact time as short as possible and jump as constantly as possible”. The software of the force plate automatically detects and eliminates any hop(s) with heel contact. We extracted the peak force (N) as the highest force value obtained during all valid hops of each set of 10 hops. The average contact time (ACT, s) was calculated as the mean of the contact time of all valid hops in each set of 10 repetitions. These two values (ACT and peak force) were extracted and used for statistical analyses as the mean of all two trials (Avg), the highest peak force value (Hv), and the lowest value for contact time (Lv).

### 2.7. Statistics

Descriptive statistics were used to calculate means and standard deviations (SD) for each testing session for all tests and for both the average of all values in a session (Avg) and the highest value in a session (Hv) for KE MVC, HG MVC, CMJ peak power and jump height, and reactive hops’ peak force, and the lowest value (Lv) per session for the reactive hops’ ACT. All data were tested for normality with the Shapiro–Wilk test and for homogeneity with Levene’s test. Changes in response to time were assessed with repeated measure analyses of variance (rmANOVA), using time (sessions one, two, and three, in pairs) as a repeated measure to determine any systematic bias [3,4]. Reliability and sensitivity statistics for each test were calculated for the Avg and Hv or LV for all three sessions together and in pairs (S1–S2; S1–S3; S2–S3) to determine which of the two gives better results.

Reliability has been classified as absolute or relative [5]. Relative reliability refers to the consistency between measurements. The intraclass correlation coefficient (ICC) is a measurement of relative reliability, and it reflects the degree of consistency and agreement between two or more variables [3]. According to the recommendations given by Koo and Li [35], the ICC and its corresponding 95% confidence intervals (95% CI) were calculated as a 2-way mixed-effects model, absolute agreement definition, and single measurement (2,1) for the Hv and Lv, and mean (2, *k*) for the Avg. These same authors set the ICC values as <0.50 for poor reliability, 0.50–0.75 for moderate reliability, 0.75–0.90 for good reliability, and >0.90 for excellent reliability. Absolute reliability refers to the inter-subject variation in the repeated measures [3,36]. The coefficient of variation (CV) is a measurement of absolute reliability. It estimates the measurement’s error considering the within-subject variation, and it is normally expressed as a percentage of its mean [3], which makes it easy to compare two similar tests or different populations [6]. The CV was calculated as $\frac{SD}{mean}\times 100$ [3,4]. An appropriate and small CV was set to <10% [4].

Sensitivity was measured for all tests as the between sessions’ standard error of measurement (SEm), smallest worthwhile change (SWC), and minimal difference (MD, also known as the smallest detectable difference). SEm indicates the error or noise of a measurement [37]. It is, therefore, useful to determine where exactly the true value of a subject lies. [2]. When compared to the SWC, the SEm is able to determine how easy it will be to notice a change in performance with a test. It can also be used to estimate sample sizes for intervention studies since the magnitude of the error directly affects the change in the mean [2]. SEm was calculated as $SD\left(pooled\right)\times \sqrt{1-ICC}$ [2,4,37] and its percentage representation as $\frac{SEm}{mean\left(pooled\right)}\times 100$ [2]. The SWC was calculated as $SD\left(pooled\right)\times 0.2$ [38]. The MD refers to the difference in two measurements that must be seen in order to qualify as meaningful or real [37], meaning a difference that is larger than the measurement error. The threshold to determine a real change in every measurement was estimated with the MD. The latter was calculated as SEm×1.96×√2, and its representation as a percentage of the mean (MD%) as $\frac{MD}{mean\left(pooled\right)}\times 100$ [37].

All analyses were executed in the statistical environment R version 4.2.0 (R Foundation for Statistical Computing, Vienna, Austria) [39]. We used the packages *rstatix* version 0.7.0 [40] for descriptive statistics and ANOVA calculations, *rio* version 0.5.29 [41] for data import and export, *MASS* [42] for normality tests, *car* [43] for homogeneity tests, and *irr* version 0.84.1 [44] for ICC. Group data are presented as means ± SD, and the level of significance was set at 0.05.

## 3. Results

The results of the Shapiro–Wilk test determined that all data were normally distributed. Levene’s test showed homogeneity in the data. All results for the reliability of the KE MVC and HG MVC are presented in Table 2, and for the CMJ, in Table 3. None of the rmANOVA tests were significant, indicating no systematic bias between test days. According to the ICC test, all comparisons of the different sessions (all three or paired across sessions) presented excellent reliability (ICC > 0.90) and a small within-subject variability or typical error for both the Avg and the Hv results (CV 2.2–6.7%). All results for the sensitivity for MVC of HG and KE are presented in Table 4 and for CMJ in Table 5. For all comparisons for these tests, the SEm < SWC, meaning that they are sensitive and thus able to detect meaningful changes in performance. Moreover, the expected changes in performance to be considered significant are, on average, MVC KE 12.1% (Avg) and 16.9% (Hv), HG MVC 8.4% (Avg) and 10.5% (Hv), CMJ height 6.1% (Avg) and 10.4% (Hv), and CMJ power 5.5% (Avg) and 13.4% (Hv).

All data for the reliability of reactive hops can be found in Table 6, and for sensitivity, in Table 7. The results of the rmANOVA showed a significant difference between sessions for peak force for S1–S3 (*p* < 0.001 for Avg and *p* = 0.006 for Hv) and S2–S3 (*p* < 0.005 for Avg and *p* = 0.002 for Hv), meaning an improvement with time was found between the first two sessions and the third one. The mean average difference for the results between S1–S2 was −3.1% (Avg) and −2.3% (Hv), between S1–S3 was −9.7% (Avg) and −7.8% (Hv), and between S2–S3 was −6.6% (Avg) and −5.5% (Hv). The ICC results over 0.90 for all comparisons show excellent reliability for this variable and the low CV (<10%), a small within-subject variability. Furthermore, the test seems to be sensitive to changes in performance if the Avg values are used but not the Hv. The expected changes in performance to be considered meaningful for this variable are, on average, 17.5% for the Avg and 18.6% for Hv. For the ACT of reactive hops, no systematic errors were found (i.e., all rmANOVA > 0.05). Both the Avg and the Lv result for the ACT present a poor to moderate reliability (ICC 0.256–0.667) and a small within-subject variability (CV < 10%). According to the analyses applied to the data, this variable is also not sensitive to small changes in performance (SEm > SWC), and the expected meaningful changes in performance are estimated to be, on average, ≥25.6% for the Avg and ≥19.2% for the Lv.

## 4. Discussion

This single-group, repeated-measures study aimed to evaluate the reliability and sensitivity of a set of common power and strength tests in a healthy and adult population over a period of nine weeks. To this end, we measured the MVC of the KE and HG, CMJ and reactive hops during three different sessions, first with an interval of one week and then for one of eight weeks. For most of the selected measurement variables, this study is the first to have applied a longer interval (i.e., more than one week) between testing sessions. For all statistical comparisons, we used the best performance value of a session (i.e., Hv or Lv, depending on the test) of every measurement, as well as the mean of all trials (Avg), to determine which of these two produced more trustworthy results. The study’s main findings were that MVC of the KE and HG, as well as CMJ, present very high reliability and sensitivity, the peak force during reactive hops is highly reliable but is only sensitive to changes in performance when the Avg results are used, and ACT during reactive hops is not a sensitive or reliable variable. Reliability and sensitivity are better for most measurements when the Avg results are used instead of the best performance value of a session (i.e., Hv or Lv).

### 4.1. Isometric Leg Strength

The results for the KE MVC on the ISOMED-2000 demonstrated this measurement to have an excellent relative (ICC 0.964–0.988) and absolute reliability (CV 4.9–6.7%, Table 2) and to be very sensitive to small changes in performance (SEm < SWC, SEm 3.8–6.6%, Table 4). There were also very small differences between the Hv and Avg results, meaning that either of them can be used and will produce trustworthy analyses. Nevertheless, not only for this performance test but for the others as well, all statistical variables improved with the use of the Avg results instead of the Hv. This is probably due to the fact that by averaging results across trials, the variability between subjects, especially in cases where extreme results are present, is decreased. Therefore, the average of trials could be a more appropriate result for a more diverse sample of participants. Several previous studies have examined the reliability of this measurement in different testing devices [45]. To the knowledge of the present authors, only one study has tested this on the ISOMED-2000, although with a short interval between testing sessions. Dirnberger et al. [28] tested participants three times (the second after 48 h and the third after 72 h) and obtained ICCs of 0.966–0.969 and SEm of 9%. The results of our study corroborate these findings and contribute further to them as they determined that this test’s reliability and sensitivity are improved by the use of the average of trials instead of the best performance value, but also does not require a familiarization session. This is probably because the specific warm-up protocol normally performed on the device serves this purpose, but also because such a device’s ability to produce reliable and sensitive results does not seem to be affected by a longer interval between measurements (e.g., eight weeks).

### 4.2. Handgrip Strength

The Jamar^®^ handgrip dynamometer is considered to be the gold standard device to test for maximal handgrip strength, and it provides the largest amount of normative data. Correspondingly, several studies have evaluated its reliability, but there are numerous differences in protocols and statistical tests applied to the data, and this, in turn, produces different results [46]. In our study, the HG MVC tested with this device resulted in excellent reliability, both relative (ICC 0.969–0.995) and absolute (CV 2.2–4.4%, Table 2). These results agree with those of previous studies using the same measuring device and similar statistics. In a population of 5–80 years old and an interval between measurements of 1 to 90 days (mean of 31 days), Hogrel [30] obtained an ICC value of 0.947 and SEm of 7.7%. Considering the highly variable sample of subjects and time between retest sessions, the results are strongly reliable. Moreover, Bohannon and Schaubert [24], working with community-dwelling elders at an interval of 12 weeks, obtained ICC values of 0.912–0.954 in a single trial per session test. Although the test was carried out with a different population, the reliability for a long interval between measurements was also excellent. Consequently, the results of our study confirm that the reliability of this upper body strength test seems to be constant even when the interval between measurements is longer than just one week. Moreover, the outcomes of our study also proved this device to be sensitive to small changes in performance, with significant changes ranging from 4.8–12.9% (Table 4, i.e., a difference of about 2.2 kg in handgrip strength can represent a real change in performance when the average of trials is used). Nevertheless, it should be noted that MD values double when the interval between measurements is more than one week. For instance, for the Avg results, MD between S1–S2 corresponds to 4.8%, in comparison to 10.8% and 10.4% for S1–S3 and S2–S3, respectively. This finding not only emphasizes the effect that time may have on the results but also that, for this measurement, the use of a familiarization session does not improve its sensitivity or reliability.

### 4.3. Countermovement Jumps

The results of the present study demonstrated very high reliability for both jump height and jump peak power, either for the Avg or the Hv between sessions (Table 3). Previous studies have reported ICC values of 0.87–0.99 for jump height [29,32,34,47] and 0.96–0.98 for jump power [29,34]. The results of the present study are, therefore, in line with previous investigations and corroborate the high reliability of this particular test. Nevertheless, only the study by Moir et al. [32] applied an interval between sessions longer than one week (i.e., four weeks) and obtained an ICC of 0.89 jump height. Consequently, to our knowledge, our study is the first one to prove that the jump height and peak power of CMJ possess a very high test–retest reliability for a longer period between sessions. For both CMJ variables, there were no significant differences between sessions (no systematic bias), and there were very small differences between the paired comparisons, meaning that the use of a familiarization session is not necessary for this type of test. Other researchers like Moir et al. [32] have also reached similar conclusions. Moreover, the error present in the results of this measurement is small (i.e., SEm% 1.8–4.2% for jump height and 1.7–4.3% for peak power, Table 5), and both the jump height and the jump peak power are sensitive variables (SEm < SWC). In other words, the CMJ test can measure very small and meaningful changes in performance (e.g., a difference of about 6% or 2.7 cm in jump height can represent a real change in performance when the average of trials is used). Other authors, like Thomas et al. [34], have found similar MDs in an adolescent athlete population (i.e., 7%), which not only corroborates the results of our study but also confirms the fact that the nature of MD as a statistical test allows comparisons across populations [37].

### 4.4. Reactive Hops

According to the results for the peak force during reactive hops, this test presents excellent relative (ICC > 0.90) and absolute reliability (CV < 8%, Table 6), and it is sensitive to changes in performance if the Avg results are used (Table 7). These results are especially important given that, so far, this is the only study to have proven the test–retest reliability in a period longer than a week for reactive hops. Moreover, a previous reliability study for this variable by Veilleux et al. [33] reported an ICC of 0.82 and a CV of 8.7% using the same device as in our study (i.e., Leonardo GRFP). When applying the same comparison as they had (i.e., 10 hops, two sessions, one week apart, and the Hv or highest result), we obtained a higher ICC (0.94) and lower CV (5.6%). The reasons for this difference could be, first, the fact that the authors calculated the ICC using a different model than ours (i.e., consistency definition and single measurement (3, 1)) which no doubt yields different results [35]; second, that the variances per se of these two tests are context-specific and sensitive to between-subjects variability [37]; and third, that the authors used the relative peak force and not the absolute, as we did. These inherent methodological components are not only part of the comparison of the two data sets (i.e., ours and that of Veilleux et al. [33]) but of any other similar reliability study comparison, and they consequently serve as an example of the importance of taking these into consideration when assessing a performance test and drawing conclusions from different measurement protocols.

In a performance protocol, familiarization with the task to be completed is a key factor to be considered. Nevertheless, not all performance tests benefit from such familiarization, and additionally, other factors, like level of expertise, also play a role [6]. According to the results of this study, the reliability of the data obtained during reactive hops might be increased by the inclusion of a familiarization session. For example, for the Avg peak force, even if the overall reliability is excellent (ICC 0.949–0.975, Table 6), when looking at the confidence intervals, the ICC for S1-S3 lies between 0.629–0.986, and for S2-S3, between 0.830–0.989. This gives the impression that when there is an initial familiarization session (i.e., S1), the variation in the results between the second and the third session is smaller and, therefore, more reliable. This is also supported by the CV results (a lower within-subject variation for S2–S3, 5.6%, than for S1–S3, 7.8%), and the lower average difference between S2–S3 (−6.6% for Avg and −5.5% for Hv) in comparison to S1–S3 (−9.7% for Avg and −7.8% for Hv). The reason for this test requiring a familiarization session might be due to the motor complexity in the nature of jumps, especially this particular type of jump [48], and the importance of it being properly executed to produce reliable results. This can be supported by the change in the mean that was observed with the significant rmANOVA. According to Hopkins [2], the change in the mean between two measurements is in itself a test of reliability. Additionally, other authors, like Markovic et al. [7], have had similar findings in the performance of horizontal jumps. They postulated that given the complexity of the task’s motor structure, at least one practice trial must be conducted to avoid the motor learning effect. In our case, and according to our findings, we would recommend a complete session where subjects can familiarize themselves with the proper technique and, therefore, prevent learning effects from influencing the data and to decrease measurement error. This finding is especially important for long-duration interventions such as those we intended to replicate in the present study, where the effect of time can produce a higher measurement error and alter the results.

Moreover, ground contact time (GCT) during reactive hopping is not in itself a performance measurement, but it has been used to determine other performance parameters. For example, it can be combined with flight time and body mass to calculate vertical stiffness during hopping [49,50], which is a measurement of the function of the stretch-shortening cycle [48]. Moreover, GCT during plyometric exercises is used as a reference parameter to determine which type of SSC is being used (i.e., fast or slow), an important variable to consider depending on the specific performance improvement that is being pursued [51]. For instance, GCTs > 0.25 s should not be considered appropriate for reactive hopping, as it is a fast SSC type of movement [52]. Since it is used to calculate other parameters, determining its reliability is essential. Several studies have researched the reliability of variables using GCT as part of the calculations [26,27,31] or using other jumping tests (e.g., depth jumps) [52,53,54,55]. To the present authors’ knowledge, very few studies have determined the reliability of only GCT in reactive hopping. For instance, Lloyd and colleagues [48] found CVs of 30–36% in total contact time in adolescent subjects, and Choukou et al. [25] an ICC 95% CI of 0.88–0.93 but with the use of an accelerometric system and not a force plate. The results of our study do not confirm these previous works. This might be due to methodological differences inherent to the type of population and to the measurement equipment used in each. Nevertheless, according to our data for the ACT, ICC values of all comparisons are not considered to possess relative reliability (ICC 0.256–0.667), and contrary to the peak force, its reliability and sensitivity do not seem to improve with the inclusion of a familiarization session. Given our findings, the use of ACT as a variable to evaluate performance, even as part of the calculations of another variable, would not be recommended.

The present study’s findings should be evaluated with one limitation. Although we asked the participants to maintain throughout the study their normal levels of physical activity, we did not control with any measurement if they had complied with this request.

## 5. Conclusions

According to the findings of our study, the isometric KE and HG MVC, and the CMJ test, possess very high reliability and are also sensitive measurements. Peak force during reactive hops is a reliable measurement, and it is a sensitive test if the Avg results are used. Additionally, reactive hops would benefit from a familiarization session when the peak force is the used variable, as it would reduce measurement error and produce more consistent results. In contrast, ACT during reactive hops has poor to moderate reliability and is not sensitive to changes in performance. In general, all tests benefit from the use of the average of trials (Avg) instead of the best performance value in a session (i.e., Hv or Lv) to produce more consistent results with lower measurement error. From an applied perspective, the results of the present paper can help sports scientists, researchers, coaches, and practitioners not only to evaluate the reliability of the tests used to measure strength and power but also to estimate error and sample size in intervention studies and determine how big the change in the performance of a test should be to qualify as meaningful and not as a measurement error.

## Figures and Tables

**Table 1:** Anthropometric characteristics of the population. Data are presented as mean ± standard deviation of all variables.

	Gender		Total
	Female	Male	
*n*	7	10	17
Age [years]	23.1 ± 1.1	24.9 ± 2.6	24.2 ± 2.2
Height [cm]	1.65 ± 0.1	1.82 ± 0.1	1.75 ± 0.1
Weight [kg]	57.9 ± 7.1	76.1 ± 13.1	68.6 ± 14.2
BMI [kg/m^2^]	21.2 ± 1.7	22.8 ± 2.8	22.1 ± 2.5

**Table 2:** Between session reliability for measurements of strength.

	S1	S2	S3	All Sessions		S1–S2		S1–S3		S2–S3	
	Mean (±SD)			ICC (95% CI)	CV%	ICC (95% CI)	CV%	ICC (95% CI)	CV%	ICC (95% CI)	CV%
**Knee extension MVC (Nm)**											
Avg	256.2 (±90)	252.9 (±95)	244.6 (±87)	0.988 (0.973–0.995)	6.2	0.988 (0.970–0.996)	5.1	0.976 (0.929–0.992)	6.7	0.981 (0.949–0.993)	5.3
Hv	262.2 (±90)	258.8 (±98)	253.6 (±92)	0.971 (0.937–0.989)	6.1	0.975 (0.937–0.991)	4.9	0.964 (0.904–0.987)	6.3	0.972 (0.925–0.990)	5.4
**Handgrip MVC (kg)**											
Avg	45.5 (±12)	45.9 (±12)	46.5 (±13)	0.990 (0.977–0.996)	3.9	0.995 (0.987–0.998)	2.2	0.979 (0.942–0.992)	3.7	0.980 (0.947–0.993)	4.4
Hv	47.7 (±12)	47.9 (±12)	48.4 (±14)	0.978 (0.952–0.991)	3.4	0.991 (0.977–0.997)	2.2	0.975 (0.933–0.991)	3.1	0.969 (0.917–0.988)	3.9

Descriptive (mean ± SD), reliability (ICC, CV%) for all measurements of strength (knee extension and handgrip MVC). Avg = average of all trials in a session; Hv: highest value in a session; SD = standard deviation, S1 = session 1, S2 = session 2, S3 = session 3, ICC = intraclass correlation coefficient, 95% CI = 95% confidence intervals, CV% = coefficient of variation.

**Table 3:** Between session reliability for countermovement jump test.

	S1	S2	S3	All Sessions		S1–S2		S1–S3		S2–S3	
	Mean (±SD)			ICC (95% CI)	CV%	ICC (95% CI)	CV%	ICC (95% CI)	CV%	ICC (95% CI)	CV%
**Jump height (cm)**											
Avg	44.2 (±11)	44.0 (±10)	44.7 (±10)	0.994 (0.986−0.998)	3.1	0.990 (0.974−0.996)	2.6	0.989 (0.970−0.996)	3.0	0.991 (0.974−0.997)	2.6
Hv	45.6 (±11)	45.9 (±10)	46.3 (11)	0.973 (0.942−0.989)	3.3	0.980 (0.947−0.992)	2.4	0.968 (0.917−0.988)	3.3	0.968 (0.916−0.988)	3.2
**Peak power (W/kg)**											
Avg	46.7 (±10)	45.9 (±10)	46.7 (±10)	0.994 (0.986−0.997)	2.8	0.991 (0.969−0.997)	2.2	0.991 (0.975−0.997)	2.6	0.988 (0.966−0.996)	2.7
Hv	48.0 (±11)	47.4 (±10)	47.8 (±10)	0.965 (0.923−0.986)	3.2	0.962 (0.904−0.986)	2.8	0.976 (0.935−0.991)	2.6	0.955 (0.882−0.984)	3.2

Descriptive (mean ± SD), reliability (ICC, CV%) for all variables for the countermovement jump test. Avg = average of all trials in a session; Hv: highest value in a session; SD = standard deviation, S1 = session 1, S2 = session 2, S3 = session 3, ICC = intraclass correlation coefficient, 95% CI = 95% confidence intervals, CV% = coefficient of variation.

**Table 4:** Between session sensitivity for measurements of strength.

	All Sessions			S1–S2			S1–S3			S2–S3		
	SEm (SEm%)	SWC	MD (MD%)	SEm (SEm%)	SWC	MD (MD%)	SEm (SEm%)	SWC	MD (MD%)	SEm (SEm%)	SWC	MD (MD%)
**Knee extension MVC (Nm)**												
Avg	9.6 (3.8%)	17.7	26.7 (10.6%)	9.8 (3.8%)	18.2	27.0 (10.6%)	13.4 (5.4%)	17.4	37.2 (14.8%)	12.2 (4.9%)	17.9	33.7 (13.6%)
Hv	15.6 (6%)	18.3	43.1 (16.7%)	14.5 (5.6%)	18.5	40.3 (15.5%)	17.0 (6.6%)	18.0	47.0 (18.2%)	15.8 (6.2%)	18.7	43.8 (17.1%)
**Handgrip MVC (kg)**												
Avg	1.2 (2.7%)	2.4	3.4 (7.4%)	0.8 (1.7%)	2.3	2.2 (4.8%)	1.8 (3.9%)	2.5	5.0 (10.8%)	1.7 (3.7%)	2.5	4.8 (10.4%)
Hv	1.8 (3.8%)	2.5	5.1 (10.7%)	1.1 (2.4%)	2.4	3.1 (6.6%)	2.0 (4.3%)	2.6	5.7 (11.8%)	2.2 (4.7%)	2.5	6.2 (12.9%)

Sensitivity tests (SEm, SWC and MD) for all measurements of strength (knee extension and handgrip MVC). Avg = average of all trials in a session; Hv: highest value in a session; S1 = session 1, S2 = session 2, S3 = session 3, SEm = standard error of measurement, SEm% = SEm as a percentage of the mean, SWC = smallest worthwhile change, MD = minimal difference, MD% = MD as a percentage of the mean.

**Table 5:** Between session sensitivity for countermovement jump test.

	All Sessions			S1–S2			S1–S3			S2–S3		
	SEm (SEm%)	SWC	MD (MD%)	SEm (SEm%)	SWC	MD (MD%)	SEm (SEm%)	SWC	MD (MD%)	SEm (SEm%)	SWC	MD (MD%)
**Jump height (cm)**												
Avg	0.8 (1.8%)	2.0	2.2 (5.1%)	1.0 (2.3%)	2.1	2.9 (6.5%)	1.1 (2.5%)	2.1	3.0 (6.8%)	1.0 (2.2%)	2.0	2.7 (6.1%)
Hv	1.7 (2.7%)	2.1	4.7 (10.3%)	1.5 (3.2%)	2.1	4.1 (8.9%)	1.9 (4.2%)	2.1	5.3 (11.5%)	1.8 (4%)	2.1	5.1 (11%)
**Peak power (W/kg)**												
Avg	0.8 (1.7%)	1.9	2.1 (4.5%)	0.9 (2%)	2.0	2.6 (5.6%)	0.9 (2%)	1.9	2.6 (5.5%)	1.0 (2.2%)	1.9	2.9 (6.2%)
Hv	1.9 (3.9%)	2.0	5.2 (10.8%)	2.0 (4.1%)	2.0	5.4 (11.4%)	1.6 (3.3%)	2.0	4.3 (9.1%)	2.0 (4.3%)	1.9	5.7 (11.9%)

Sensitivity tests (SEm, SWC, and MD) for all variables for the countermovement jump test. Avg = average of all trials in a session; Hv: highest value in a session; S1 = session 1, S2 = session 2, S3 = session 3, SEm = standard error of measurement, SEm% = SEm as a percentage of the mean, SWC = smallest worthwhile change, MD = minimal difference, MD% = MD as a percentage of the mean.

**Table 6:** Between session reliability for reactive hops.

	S1	S2	S3	All Sessions		S1–S2		S1–S3		S2–S3	
	Mean (±SD)			ICC (95% CI)	CV%	ICC (95% CI)	CV%	ICC (95% CI)	CV%	ICC (95% CI)	CV%
**Peak force (kN)**											
Avg	3.48 (±1)	3.58 (±1)	3.81 (±1) #, &	0.975 (0.925–0.991)	7.1	0.972 (0.926–0.989)	5.7	0.949 (0.629–0.986)	7.8	0.965 (0.830–0.989)	5.6
Hv	3.69 (±1)	3.74 (±1)	3.95 (±1) #, &	0.942 (0.859–0.978)	6.4	0.94 (0.848–0.977)	5.6	0.926 (0.703–0.976)	6.7	0.956 (0.748–0.987)	4.9
**Average contact time (s)**											
Avg	0.182 (±0.02)	0.171 (±0.02)	0.180 (±0.02)	0.526 (−0.031–0.811)	8.5	0.659 (0.090–0.872)	6.0	0.32 (−1.014–0.760)	8.9	0.256 (−0.909–0.723)	7.9
Lv	0.168 (±0.02)	0.165 (±0.02)	0.169 (±0.02)	0.536 (0.250–0.776)	6.2	0.667 (0.316–0.859)	4.5	0.511 (0.039–0.792)	5.7	0.457 (−0.006–0.761)	6.4

Descriptive (mean ± SD), reliability (ICC, CV%) for all variables for reactive hops. Avg = average of all trials in a session; Hv: highest value in a session; Lv: lowest value in a session; SD = standard deviation, S1 = session 1, S2 = session 2, S3 = session 3, # = refers to a significant difference (*p* < 0.05) in the rmANOVA between sessions 1–3, & = refers to a significant difference (*p* < 0.05) in the rmANOVA between sessions 2–3, ICC = intraclass correlation coefficient, 95% CI = 95% confidence intervals, CV% = coefficient of variation.

**Table 7:** Between session sensitivity for reactive hops.

	All Sessions			S1–S2			S1–S3			S2–S3		
	SEm (SEm%)	SWC	MD (MD%)	SEm (SEm%)	SWC	MD (MD%)	SEm (SEm%)	SWC	MD (MD%)	SEm (SEm%)	SWC	MD (MD%)
**Peak force (kN)**												
Avg	0.16 (4.5%)	0.20	0.45 (12.5%)	0.17 (4.8%)	0.20	0.47 (13.4%)	0.23 (6.4%)	0.21	0.65 (17.8%)	0.19 (5.1%)	0.20	0.53 (14.3%)
Hv	0.25 (6.7%)	0.21	0.70 (18.4%)	0.26 (6.9%)	0.21	0.71 (19.2%)	0.30 (7.7%)	0.22	0.82 (21.4%)	0.21 (5.6%)	0.20	0.59 (15.4%)
**Average contact time (s)**												
Avg	0.015 (8.6%)	0.004	0.042 (23.8%)	0.012 (6.9%)	0.004	0.034 (19.1%)	0.019 (10.8%)	0.005	0.054 (29.9%)	0.019 (10.7%)	0.004	0.052 (29.8%)
Lv	0.012 (7.0%)	0.003	0.032 (19.3%)	0.009 (5.6%)	0.003	0.026 (15.6%)	0.012 (7%)	0.003	0.033 (19.4%)	0.013 (8.1%)	0.004	0.037 (22.4%)

Sensitivity tests (SEm, SWC, and MD) for all variables for reactive hops. Avg = average of all trials in a session; Hv: highest value in a session; Lv: lowest value in a session; S1 = session 1, S2 = session 2, S3 = session 3, SEm = standard error of measurement, SEm% = SEm as a percentage of the mean, SWC = smallest worthwhile change, MD = minimal difference, MD% = MD as a percentage of the mean.4.3. Countermovement jump.

## Data Availability

All relevant data are within the manuscript. The datasets generated during and/or analyzed during the current study are available from the corresponding author upon reasonable request.
